# Interstitial lung disease associated with ALK inhibitors and risk factors: an updated comparative pharmacovigilance analysis

**DOI:** 10.3389/fphar.2024.1361443

**Published:** 2024-09-27

**Authors:** Junli Dong, Lulu Li, Tiying Deng, Haibin Song, Shaohui Zhang, Minyu Zhong

**Affiliations:** ^1^ Department of Pharmacy, Traditional Chinese and Western Medicine Hospital of Wuhan, Tongji Medical College, Huazhong University of Science and Technology, Wuhan, China; ^2^ Department of Pharmacy, Wuhan No.1 hospital, Wuhan, China; ^3^ Department of Oncology, Traditional Chinese and Western Medicine Hospital of Wuhan, Tongji Medical College, Huazhong University of Science and Technology, Wuhan, China; ^4^ Department of Oncology, Wuhan No.1 hospital, Wuhan, China

**Keywords:** FDA adverse event reporting system, ALK TKIs, interstitial lung disease, pharmacovigilance, adverse events

## Abstract

**Background:**

Inhibitors of the anaplastic lymphoma kinase (ALK) gene mutation are first-line treatments in patients with ALK-positive lung cancer. The FDA label warns of the risk of interstitial lung disease (ILD) in patients receiving ALK TKIs. However, ILD associated with ALK TKIs is not fully understood. The aim of this study was to characterize the features of ALK TKI-related ILD and to explore risk factors for ALK TKI-related ILD.

**Methods:**

FDA’s Adverse Event Reporting System (FAERS) reports from 2011 Q1 to 2023 Q2 were extracted and combined. Standardized MedDRA queries (SMQs) were used to search for AEs at the preferred term (PT) level. Four algorithms were employed to quantify the signals of ILD associated with ALK TKIs. The risk of ILD was further analyzed using logistic regression.

**Results:**

A total of 20,064 reports of ALK TKIs and 640 (3.2%) reports of ILD AEs were extracted. Significant disproportionality was detected in all five ALK TKIs. Interstitial lung disease and pneumonitis were the most common lung toxicities induced by ALK TKIs. Results of further analyses revealed a different spectrum of lung toxicity among the various TKIs. The median time to onset of ILD related to ALK TKIs was 53 days (Q1:12, Q3:209), and more than 70% of AEs occurred within the first 2 months. Logistic regression analysis and risk prediction model both showed that different ALK TKIs and their combination with PPIs, amlodipine, and magnesium oxide were independent risk factors for ILD (*p*<0.05).

**Conclusion:**

ALK TKIs have different safety profiles regarding lung toxicity, which normally occurs within the first 2 months. Administration in combination with PPIs, amlodipine, and magnesium oxide significantly increases the risk of ILD. These results provide risk prediction for ILD related to ALK TKIs and support pharmacovigilance to promote safe prescribing in oncology.

## Introduction

Non-small cell lung cancer (NSCLC) is the leading cause of cancer-related deaths globally (Sung et al., 2021). A rearranged anaplastic lymphoma kinase (ALK) gene/fusion is a unique biomarker in NSCLC patients, presenting in approximately 3%–7% of cases (Horn and Pao, 2009). Over the past decade, the emergence of ALK tyrosine kinase inhibitors (TKIs) has significantly transformed the treatment landscape and outcomes for ALK+ NSCLC patients (Solomon et al., 2014; Shaw et al., 2014; Peters et al., 2017; Camidge et al., 2018; Shaw et al., 2020).

In 2011, the Food and Drug Administration (FDA) approved crizotinib as the first ALK TKI for patients with ALK+ NSCLC (Crino et al., 2011; Kim et al., 2012). This multi-targeted TKI demonstrated efficacy against MET (mesenchymal–epithelial transition, a prototypical receptor tyrosine kinase, whose alterations are drivers of human cancer), ALK, and ROS1 (ROS proto-oncogene 1, a receptor tyrosine kinase, which is involved in genetic rearrangement in a variety of human cancers), ALK, and ROS1, and was proven to improve progression-free survival (PFS) compared with traditional chemotherapy (median 10.9 months vs. 7.0 months; HR 0.45; 95% CI 0.35–0.60; *p* < 0.001) in patients with ALK-positive lung cancer (Shaw et al., 2103). However, crizotinib has been widely replaced by next-generation TKIs in first-line therapy due to its poor intracranial activity and shorter PFS (Gadgeel et al., 2120; Crinò et al., 2016). Currently, second-generation ALK TKIs have been developed, including ceritinib, alectinib, and brigatinib, which address the crizotinib resistance and offer better blood–brain barrier (BBB) penetration in patients with CNS involvement (Mok et al., 2011; Ou et al., 2016; Dong-Wan et al., 2017; D Ross Camidge et al., 2018). Alectinib exhibits longer PFS than crizotinib (median 34.8 months vs. 10.9 months) (Camidge et al., 2019); however, it has also been associated with the problem of drug resistance (Gainor et al., 2016). Lorlatinib, an ATP-competitive macrocyclic small-molecule inhibitor, was developed to resolve the problem of resistant mutations that occur during treatment with the first second-generation ALK TKIs (Abbattista et al., 2019; Zou et al., 2015). To date, the FDA has approved five ALK TKIs (crizotinib, ceritinib, alectinib, brigatinib, and lorlatinib) as the first-line and follow-up treatment drugs for patients with ALK+ NSCLC (Pirker and Filipits, 2019).

Treatment with ALK TKIs is normally well-tolerated. However, the patterns and frequency of side effects differ among ALK TKIs (Omar et al., 2021), which may be a key consideration for physicians when choosing a medication. Interstitial lung disease (ILD) is a heterogeneous group of parenchymal lung diseases with high morbidity and mortality (Antoniou et al., 2014). Several risk factors for ILD also coexist, including anticancer drugs (Spagnolo et al., 2022). A system study found that ALK TKIs showed significant respiratory system toxicity, with pneumonia being the most common serious adverse event with the highest incidence (Hou et al., 2019). Recently, a report characterized interstitial pneumonitis (IP) associated with ALK TKIs in the real world and indicated a fatal risk of IP induced by ALK TKIs (Ma et al., 2023; Zhao et al., 2023). The FDA released a hazard alert on ALK TKIs for ILD; however, a comprehensive evaluation of ILD induced by five ALK TKIs and the risks of ILD was still inadequate.

Therefore, it is important to explore the clinical characteristics and risk factors of ALK TKI-induced ILD to ensure appropriate drug selection. The aim of this study was to comprehensively characterize ILD with ALK TKIs in real-world patterns by investigating the FDA’s Adverse Event Reporting System (FAERS) and to evaluate the risk of ILD. A risk model for predicting ILD with ALK TKIs was constructed using logistic regression, and risk factors were determined for medication decisions.

## Materials and methods

### Data sources

This observational, retrospective pharmacovigilance analysis was conducted using data from adverse event reports recorded in the FAERS database, which are available at https://fis.fda.gov/extensions/FPD-QDE-FAERS/FPD-QDE-FAERS.html. The data downloaded were limited to the period from 1 January 2011 to June 2023 (the most recent available data), which comprised seven tables named “DEMO,” “DRUG,” “REAC,” “OUTC,” “RPSR,” “THER,” and “INDI”. The “DEMO” tables were used in the missing value imputation and case de-duplication steps. AEs were coded according to the Medical Dictionary for Regulatory Activities Terminology (MedDRA) at the preferred term (PT) level. For signal detection, the drug was considered the primary suspect.

### Data processing

Data processing included missing value imputation, case de-duplication, and standardization (Supplementary Figure S1), with reference to the study by Nigam H. Shah et al. (Banda et al., 2016). First, a single missing value imputation was performed for four fields, including event date, age, sex, and reporter country. For other versions of the same case, the maximum demographic values from the fully populated case versions were employed to fill in single missing values. Then, a two-step de-duplication was performed. If all available cases shared identical values for case ID, initial/follow-up code, case event date, age, sex, reporter country, drug names, and outcomes, the most recent case version was retained. Based on four demographic data fields (event date, age, sex, and reporter country), data from the DEMOyyQq tables were de-duplicated and linked to DRUG, INDI, and REAC, respectively, by primaryid,. The adverse events in which ALK inhibitors were the primary suspect were included, while the cases with ages less than 18 years were excluded. Finally, data from DRUG, INDI, and REAC were standardized by using the Observational Health Data Sciences and Informatics (OHDSI) Vocabulary 5.0 and the Medical Dictionary for Regulatory Activities (MedDRA). We screened available standardized MedDRA queries (SMQs) for “Interstitial lung disease (20000042).” Associated PTs were acute interstitial pneumonitis (10066728), idiopathic interstitial pneumonia (10078268), interstitial lung abnormality (10087834), interstitial lung disease (10022611), pneumonitis (10035742), pulmonary fibrosis (10037383), etc. Supplementary Table S1 shows the full list of PTs within the ILD SMQs.

### Statistical analyses

The measurement data were characterized by median and interquartile ranges, while enumeration data were presented as numbers and percentages. The correlation between an AE and the drug was investigated by disproportionality analysis, including the reporting odds ratio (ROR), the proportional reporting ratio (PRR), the information component (IC), and the empirical Bayes geometric mean (EBGM). Odds ratios (ORs) were calculated using a logistic regression model to assess the association between potential risk factors and ILD in patients receiving ALK TKIs. Additionally, a nomogram was constructed to estimate the probability of developing ILD in NSCLC patients receiving different ALK TKIs. Each variable corresponds to a line segment with scales marked, which represents the range of values that the variable can take, and the length of the line segment reflects the contribution of that factor to the occurrence of ILD.

## Results

### Descriptive analysis

From January 2011 to June 2023, 15,656,531 reports were recorded in FAERS. After excluding duplicate cases (2,126,495) and aberrant cases with age less than 18 years (439,205), 13,090,831 reports were included in the present analysis. Of these, 20,064 AE reports were related to ALK TKIs (0.15%), including 9,130 for crizotinib, 1,929 for ceritinib, 4,673 for alectinib, 2,138 for brigatinib and 2,492 for lorlatinib (Supplementary Table S2).

AE reports of ILD among ALK TKI users accounted for 3.2% (640/20,064) of the total AEs related to ALK TKIs; of these, 45.9% were related to crizotinib, 8.0% to ceritinib, 24.5% to alectinib, 14.7% to brigatinib, and 6.9% to lorlatinib. The average age of ALK TKI users differed: for alectinib and brigatinib, it was 63 years, and the lowest mean age for ceritinib was 56 years. Comparable percentages of male and female patients were observed for all five drugs. Of note, the majority of reports were submitted by physicians with the highest percentage of reports for crizotinib (65.0%), alectinib (66.9%), brigatinib (61.7%), and lorlatinib (63.6%), or by consumers with the highest percentage of reports for ceritinib (35.3%). Hospitalization and death were recorded in 40.7% and 18.8% of cases, respectively (Table 1).

**TABLE 1:** Demographics related to ILD reported in patients receiving ALK TKIs.

	Crizotinib	Ceritinib	Alectinib	Brigatinib	Lorlatinib	*p*-Value
Total cases, n (%)	294 (45.9)	51 (8.0)	157 (24.5)	94 (14.7)	44 (6.9)	
Age (years), mean ± SD	61.9 ± 13.3	56.9 ± 14.8	63.6 ± 12.0	63.3 ± 13.1	58.9 ± 13.9	0.495
18–64	127	23	53	34	23	
65–84	108	14	60	32	12	
>85	4	0	1	2	1	
Unknown	55	14	43	26	8	
Weight (kg), mean ± SD	61.6 ± 17.2	70.2 ± 18.0	67.1 ± 19.5	64.0 ± 16.0	66.1 ± 17.1	0.211
Sex, n (%)						0.066
Male	131 (44.6)	21 (41.2)	80 (51.0)	30 (31.9)	25 (56.8)	
Female	140 (47.6)	23 (45.1)	62 (39.5)	48 (51.1)	16 (36.4)	
Unknown	23 (7.8)	7 (13.7)	15 (9.5)	16 (17.0)	3 (6.8)	
Report source, n (%)						<0.001
Physician	191 (65.0)	13 (25.5)	105 (66.9)	58 (61.7)	28 (63.6)	
Pharmacist	25 (8.5)	3 (5.9)	13 (8.3)	6 (6.4)	4 (9.1)	
Consumer	35 (11.9)	18 (35.3)	20 (12.7)	14 (14.9)	6 (13.6)	
Other	42 (14.3)	16 (31.4)	19 (12.1)	16 (17.0)	5 (11.4)	
Unknown	1 (0.3)	1 (2.0)	0 (0)	0 (0)	1 (2.3)	
Outcome						<0.001
Hospitalization	166 (35.0)	24 (40.7)	71 (53.0)	56 (55.4)	20 (33.3)	
Disability	10 (2.1)	1 (1.7)	1 (0.7)	1 (1.0)	0 (0)	
Life-threatening	51 (10.8)	6 (10.2)	13 (9.7)	7 (6.9)	6 (10.0)	
Death	98 (20.7)	11 (18.6)	18 (13.4)	15 (14.9)	14 (23.3)	
Other	149 (31.4)	17 (28.8)	31 (23.1)	22 (21.8)	20 (33.3)	

### Disproportionality analysis

As shown in Table 2, the signal of ILD was detected in all ALK TKIs. Treatment with ALK TKIs was significantly associated with ILD, with ROR (4.58, 95% CI 4.23–4.96), PRR (4.47), IC (2.15, IC025 1.99–2.33), and EBGM (4.45, 95% CI 4.16–4.75). Brigatinib reported the highest ROR for ILD (6.38, 95%CI 5.19–7.85) with alectinib (4.84, 95% CI 4.13–5.68) and crizotinib (4.71, 95%CI 4.2–5.3) following.

**TABLE 2:** Signal detection of ILD for each ALK inhibitor at the SMQ level.

Drug	ROR (95% CI)	PRR (x^2^)	IC(IC025)	EBGM(95% CI)
ALK TKIs	4.58 (4.23, 4.96)	4.47 (1723.87)	2.15 (1.99, 2.33)	4.45 (4.16, 4.75)
Crizotinib	4.71 (4.2, 5.3)	4.59 (829.53)	2.2 (1.95, 2.47)	4.58 (4.16, 5.05)
Ceritinib	3.81 (2.88, 5.03)	3.73 (102.68)	1.9 (1.44, 2.51)	3.73 (2.96, 4.71)
Alectinib	4.84 (4.13, 5.68)	4.71 (461.36)	2.23 (1.9, 2.62)	4.7 (4.12, 5.37)
Brigatinib	6.38 (5.19, 7.85)	6.15 (407.55)	2.62 (2.13, 3.22)	6.14 (5.16, 7.3)
Loratinib	2.55 (1.89, 3.44)	2.52 (40.72)	1.33 (0.99, 1.8)	2.52 (1.97, 3.24)

**FIGURE 1:**
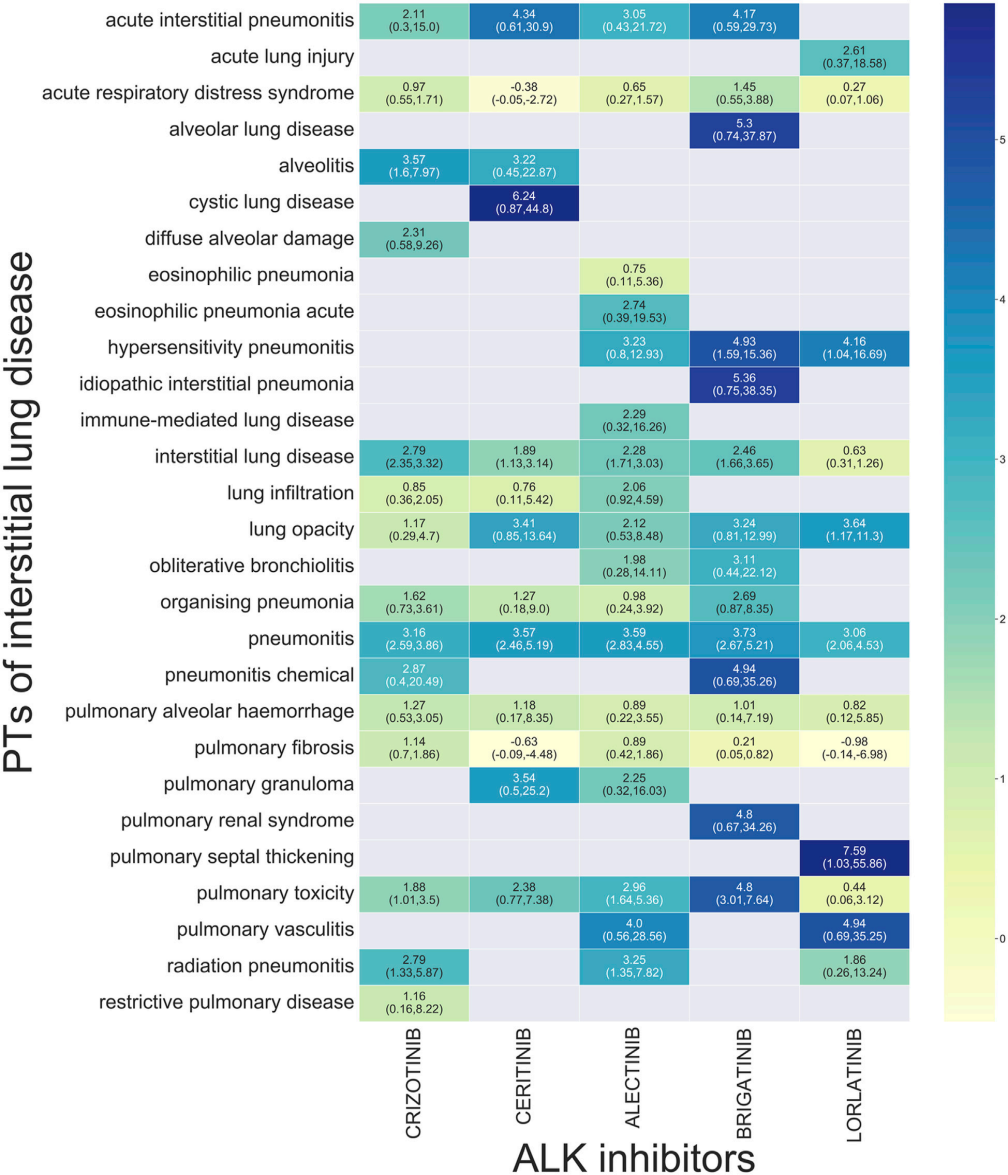
Signal profiles of ILD for each ALK inhibitor.

Disproportionality analysis for subgroups of ILD showed that 28 preferred terms (PTs) were significantly associated with ALK TKIs overall. Twelve PTs were significantly associated with crizotinib and ceritinib, and seven PTs were associated with alectinib and brigatinib. In contrast, only five PTs were associated with lorlatinib. Our analysis found a significant signal for pneumonitis for all analyzed ALK TKIs. Concerning acute interstitial pneumonitis, a significant signal was found for all ALK TKIs, with the exception of lorlatinib. Only crizotinib was associated with acute eosinophilic pneumonia and immune-mediated lung disease. Signals of alveolar lung disease, idiopathic interstitial pneumonia, and pulmonary-renal syndrome were only found for ceritinib. Only brigatinib was associated with diffuse alveolar damage. Only lorlatinib was associated with a significant ROR for acute lung injury and pulmonary septal thickening (Figure 1).

### Time of onset and cumulative dose of ALK TKI-associated ILD

Of the patients who developed ILD, 56.8% developed ILD within 4 weeks of initiating ALK TKI therapy, and 70.4% developed it within 8 weeks. For crizotinib, 56.8% and 72.3% of cases developed ILD within 4 and 8 weeks, respectively. For ceritinib, 59.1% of cases developed ILD within 8 weeks. For alectinib, 44.4% and 61.1% of cases developed ILD within 4 and 8 weeks, respectively. For brigatinib, 77.6% and 85.7% of cases developed ILD within 4 and 8 weeks, respectively. For lorlatinib, 47.1% and 64.7% of cases developed ILD within 4 and 8 weeks, respectively (Supplementary Table S3).

**FIGURE 2:**
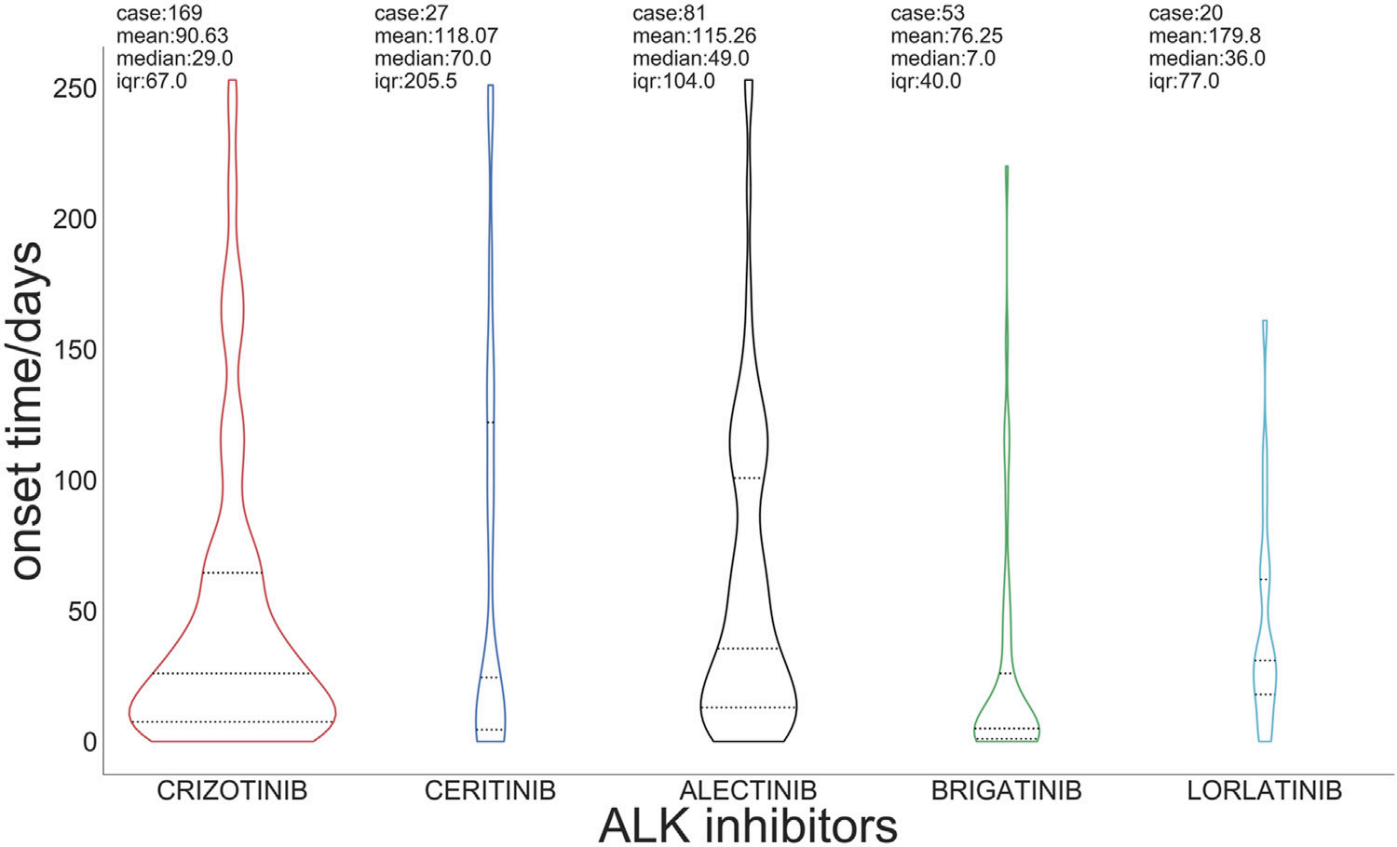
Time to onset of interstitial lung disease induced by ALK inhibitors.

Figure 2 presents the box plots and medians of the onset time of ILD with regard to ALK TKIs. The median onset time of ILD related to total ALK TKIs was 53 days (Q1:12, Q3:209). The median time of onset was 29 days for crizotinib, 70 days for ceritinib, 49 days for alectinib, 7 days for brigatinib, and 36 days for lorlatinib (Supplementary Table S3).

**FIGURE 3:**
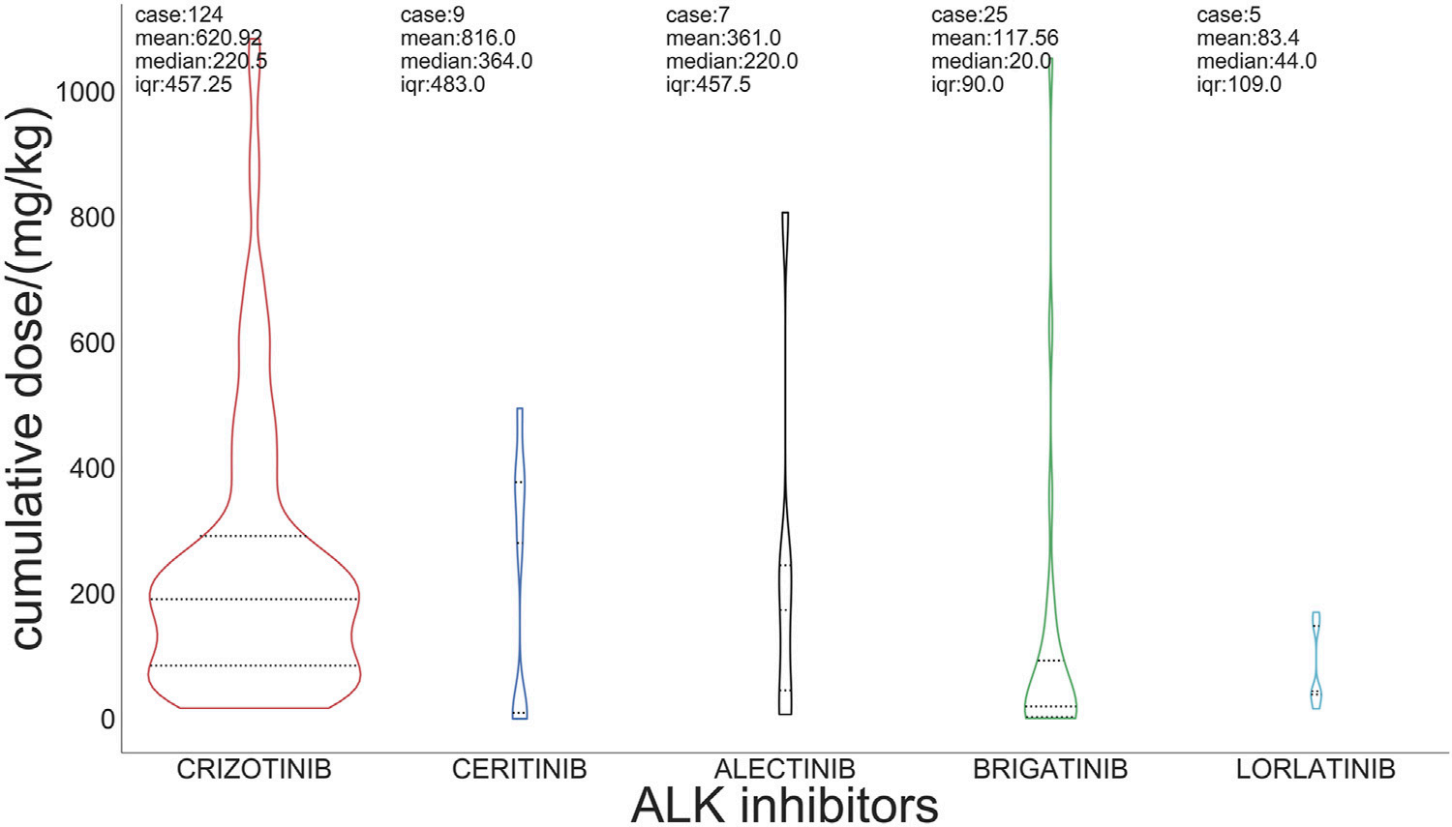
Cumulative dose of ALK inhibitors associated with ILD.

For the incidence of ILD, the median cumulative dose of crizotinib was 220 mg/kg, and the doses of ceritinib, alectinib, brigatinib, and lorlatinib were 364 mg/kg, 220 mg/kg, 20 mg/kg, and 44 mg/kg, respectively (Figure 3).

### Risk factors for developed ILD and nomogram

**FIGURE 4:**
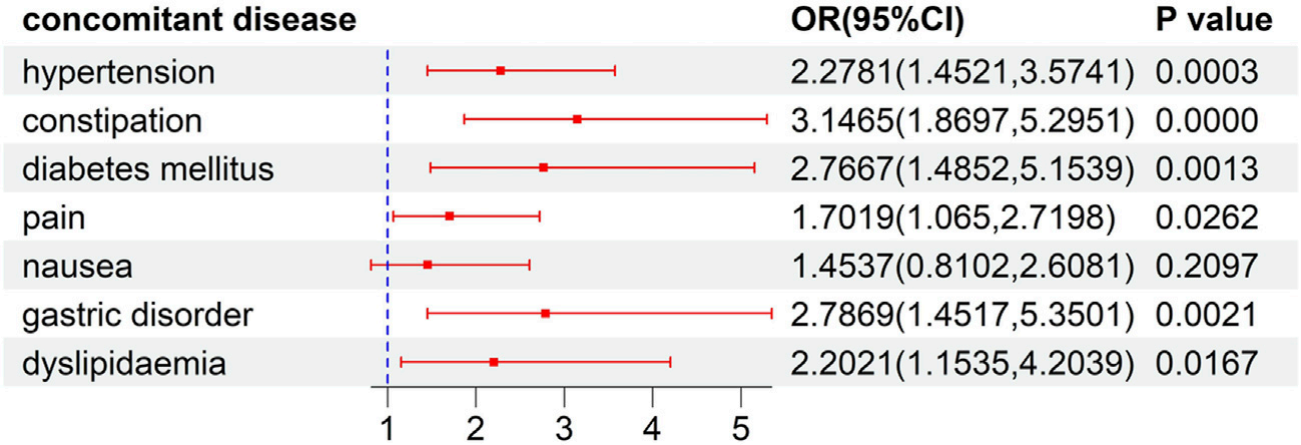
The risk of ILD induced by concurrent diseases.

**FIGURE 5:**
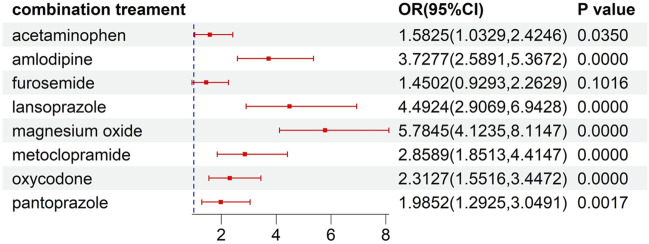
The risk of ILD induced by combination treatment.

This analysis of risk factors for developed ILD was conducted on patients receiving ALK TKI therapy. The results of univariate logistic regression, as illustrated in Supplementary Table S4, indicated that female gender, concomitant disease, and concomitant drug significantly increased the risk of the developed ILD (P<0.05). To further explore the effects of concomitant factors on the developed ILD, we observed that patients with gastric disorder, pain, diabetes mellitus, hypertension, dyslipidemia, and constipation might be at a higher risk than those without concomitant diseases (Figure 4). Meanwhile, the risks of ILD associated with ALK inhibitors in combination with acetaminophen, amlodipine, lansoprazole, magnesium oxide, metoclopramide, oxycodone, and pantoprazole were higher than the risks of ALK inhibitor monotherapy (Figure 5).

According to multivariate logistic regression analysis with adjustment for confounding variables, as shown in Table 3, amlodipine, magnesium oxide, lansoprazole, and pantoprazole had a significant effect on the development of ILD in patients receiving ALK TKIs (P<0.05).

**TABLE 3:** Multivariable logistic regression analysis of drug-induced ILD.

Characteristics	Odds ratio	95% CI	*p*-Value
Age	3.087	2.579, 3.695	0.191
Sex	2.160	1.826, 2.555	0.002
Concomitant diseases			
Hypertension	2.541	1.444, 4.474	0.809
Diabetes mellitus	4.541	2.201, 9.372	0.262
Dyslipidemia	3.080	1.420, 6.682	0.766
Gastric disorder	6.018	2.969, 12.198	0.105
Pain	3.235	1.878, 5.573	0.563
Nausea	2.157	1.113, 4.180	0.436
Constipation	2.934	1.570, 5.483	0.818
Combined drug category			
Amlodipine	10.129	6.657, 15.414	<0.001
Furosemide	2.623	1.649, 4.171	0.878
Magnesium oxide	33.730	22.511, 50.541	<0.001
Lansoprazole	9.759	6.001, 15.872	0.001
Pantoprazole	4.943	3.139, 7.783	0.043
Acetaminophen	2.489	1.555, 3.986	0.701
Oxycodone	3.548	2.265, 5.558	0.302
Metoclopramide	4.374	2.660, 7.192	0.125
Crizotinib	4.939	3.525, 6.921	0.007
Ceritinib	4.022	2.605, 6.212	0.136
Alectinib	5.409	3.789, 7.720	0.004
Brigatinib	8.086	5.490, 11.909	<0.001

**FIGURE 6:**
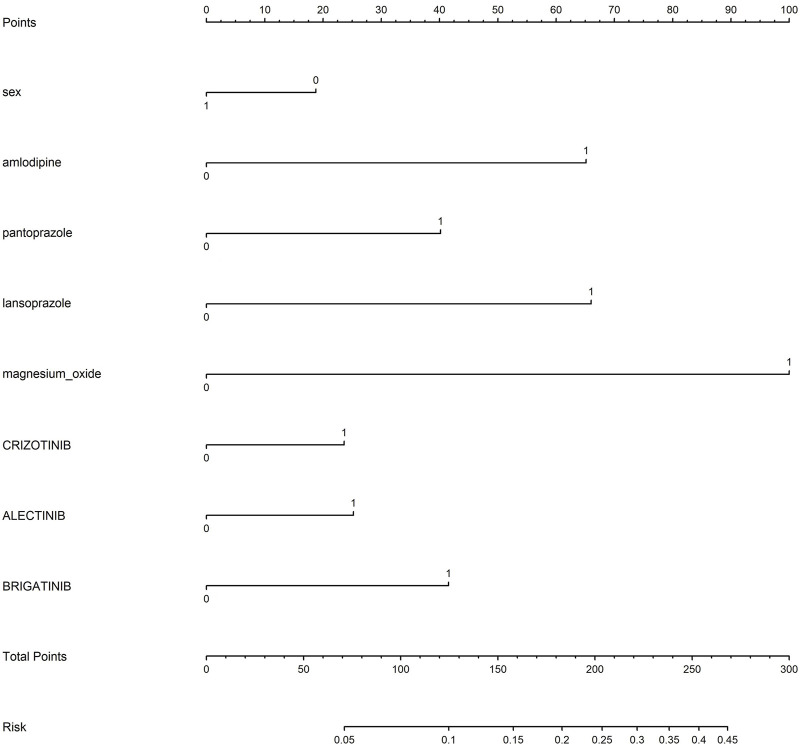
Nomogram for predicting interstitial lung disease in patients receiving ALK TKIs.

Based on the results of univariate and multivariate logistic regression, sex, concomitant diseases, and concomitant drugs were included in the nomogram model. Figure 6 represents the nomogram for predicting the risk of developing ILD in patients receiving ALK TKIs. The results suggested that women were at higher risk than men. Between the two PPIs, lansoprazole posed a greater risk of ILD than pantoprazole. Furthermore, brigatinib caused the highest risk among five ALK TKIs, whereas ceritinib and loratinib did not significantly contribute to the risk of ILD.

## Discussion

Pulmonary toxicities induced by targeted anticancer therapy are overall infrequent but potentially life-threatening (Teuwen et al., 2015). Especially combined with risk factors, the management of pulmonary toxicities can become a substantial therapeutic challenge and significantly influence the overall prognosis of cancer patients.

ALK TKI has become the standard treatment for NSCLC. However, the safety profile of each ALK TKI is different with regard to pulmonary toxicity. The retrospective studies indicated that the median age of diagnosis for ALK+ NSCLC was approximately 60 years (Auliac et al., 2017; Lim et al., 2017), and these older patients have comorbidities and polypharmacy (Decoster and Schallier, 2019), which may increase TKI-mediated toxicities during long-term treatment. Although the present study characterizes a different toxicity profile of ALK TKIs regarding ILD (Zhao et al., 2023), a comprehensive risk evaluation of ILD induced by ALK TKIs was still inadequate. Therefore, our pharmacovigilance analysis further illuminated the complex safety profile of ALK TKIs and independent risk factors by characterizing global data from the FAERS database.

ILD is a fatal but less frequently occurring category of adverse reactions. The FAERS database showed that ILD occurred in 3.2% (1.8%–4.4%) of the reports treated with ALK TKIs from 2011 to 2023. The proportion of ILD was the highest in brigatinib reports (4.4%) and lowest in lorlatinib reports (1.8%), which is consistent with the ALTA-1L clinical trials (Camidge et al., 2018) (D Ross Camidge et al., 2020) and FDA labeling (Fdalabel, 2023).

All five ALK TKIs could induce pulmonary toxicity; however, the adverse event profiles at the PT level were different between different ALK TKIs. Brigatinib was associated with the strongest and most robust disproportionality signal of ILD, which was consistent with the evidence from a systematic study (Pellegrino et al., 2018). All ALK TKIs present pneumonitis and lung opacities, but alectinib was associated with more PTs than acute eosinophilic pneumonia, and lung infiltration was not shown with other inhibitors. The mechanism of ILD induced by ALK TKI is not fully understood. Research has demonstrated that cross-reactivity with other kinases, such as EGFR, MET, and ROS1, may significantly contribute to the inhibition of normal signaling and impairment of the lung epithelium (Hwang et al., 2018; Harada et al., 2011). Gurule et al. speculated that TKI may be related to the disruption of normal epithelial tissue homeostasis and the induction of an innate inflammatory immune response (Gurule and Heasley, 2018). The mechanism underlying superimposed lung damage may be complex and must be further explored.

More than 70% of cases of ALK TKI-related ILD occurred within the first 2 months of initiating ALK TKI treatments. However, the median time to onset of ILD varied among ALK TKIs, with ceritinib exhibiting a notably longer delay, whereas brigatinib displayed the shortest time to onset. In clinical trials, early-onset pulmonary events were observed in the brigatinib group, especially within the first 3–8 days of treatment (Camidge et al., 2018). This indicates that early monitoring for pulmonary toxicity associated with brigatinib is necessary, especially for the prompt diagnosis of signs and typical symptoms such as dyspnea, hypoxia, and dry cough.

In a further investigation of factors affecting ALK TKI-related ILD, we observed a higher risk of ALK TKI-related ILD in the female group. A retrospective study showed that sex has no impact on lung toxicity in NSCLC patients exposed to ALK TKIs (Hwang et al., 2019), but another study suggested that ILD onset in patients receiving crizotinib was affected by sex in the univariate model (Gemma et al., 2019). The controversy regarding the effect of sex on ILD in ALK TKIs may be due to a variety of reasons, including variations in sampling, disparities between study cohorts, and different adjustments for confounding variables.

To date, no study has examined how coadministration with other medications influences the development of ILD with ALK TKIs. We observed that amlodipine, PPIs, and magnesium oxide were significantly associated with an increased risk of ALK TKI-related ILD. There was evidence indicating an increased risk of ILD in patients receiving lansoprazole (Kambara Kh et al., 2020; Atkins et al., 2014). According to the report by Kawamura et al., patients receiving a concomitant PPI demonstrated a higher incidence and risk of acute exacerbation of interstitial pneumonia (Kawamura et al., 2019). These studies indicate a strong correlation between PPI use and the occurrence of ILD. There was little evidence of a relationship between amlodipine, magnesium oxide, and ILD. However, amlodipine has been reported to improve the anticancer effects of gefitinib and regorafenib (Fu et al., 2022; Alandağ et al., 2022). The mechanism of the synergistic anticancer effect was that amlodipine can enhance the intracellular uptake of anticancer drugs (Li et al., 2006) as a Ca^2+^ channel blocker. Therefore, we speculated that the cytotoxicity of the targeted drug was probably potentiated by amlodipine, which resulted in an increased risk of ADR with concomitant use of amlodipine in patients receiving targeted therapy. These findings underscore the need for ongoing epidemiologic monitoring and urgent clarification through large-scale, population-based studies in addition to specialized RCTs.

A model to predict the risk of ILD after administration of ALK TKIs in NSCLC patients was developed based on univariate and multivariable regression. The risk of ILD caused by ALK inhibitors is approximately 3%, but we could observe that the risk of developing ILD increased to approximately 10% in a female lung cancer patient who was receiving treatment with brigatinib and concurrently taking amlodipine, as determined by the accumulation of individual risk factors. The results indicate that patients, particularly women receiving ALK inhibitors, need to avoid or cautiously combine the use of drugs with a higher risk of developing ILD. The predictors used in our study are available in the clinic, including sex and concomitant drugs, such as amlodipine prescribed to patients with hypertension and PPIs used by patients with gastric ulcers. The model allows physicians to assess the risk of ILD in cancer patients with concomitant drugs and make treatment decisions.

Our study has certain limitations. First, as a spontaneous reporting system, the FAERS database often contains incomplete or missing information and lacks data on population exposure. This precluded us from providing incidence rates of ADR (Bate and Evans, 2009). Furthermore, insufficient clinical features (such as body mass and patient history) and instrumental assessments (e.g., CT imaging and laboratory parameters) to support disease diagnosis, severity assessment, and prediction of potential ILD risk limit in-depth analysis and understanding of adverse events. Finally, the contribution of ALK TKIs to ILD-related deaths cannot be determined but is worth confirmation in a large-scale prospective study. Nonetheless, the results of our study highlight an increased risk of ILD associated with ALK TKIs for the treatment of NSCLC and provide a better basis for understanding potential ILD associated with ALK TKIs, which helps clinicians pay attention to risk management.

## Data Availability

The datasets presented in this study can be found in online repositories. The names of the repository/repositories and accession number(s) can be found in the article/Supplementary Material.
