# Supplementary material for: Interstitial lung disease associated with ALK inhibitors and risk factors: an updated comparative pharmacovigilance analysis
Source: Front Pharmacol. 2024 Sep 27;15:1361443. doi: 10.3389/fphar.2024.1361443 (PMC11466793; doi:10.3389/fphar.2024.1361443)
Supplement: Supplementary file 1 [file DataSheet1.docx]

Supplementary Material

Interstitial lung disease associated with anaplastic lymphoma kinase tyrosine kinase inhibitor and risk factors: an updated comparative pharmacovigilance analysis.

Junli Dong, Lulu Li, Tiying Deng, Haibin Song, Shaohui Zhang*, Minyu Zhong*

* Correspondence: Corresponding Author:

Shaohui Zhang, [zshtjmu@hotmail.com](mailto:zshtjmu@hotmail.com)

Minyu Zhong, [zmy405645073@163.com](mailto:zmy405645073@163.com)

# Supplementary Data

Supplementary data includes 1 figure and 4 tables.

# Supplementary Figures and Tables

## Supplementary Figures

**
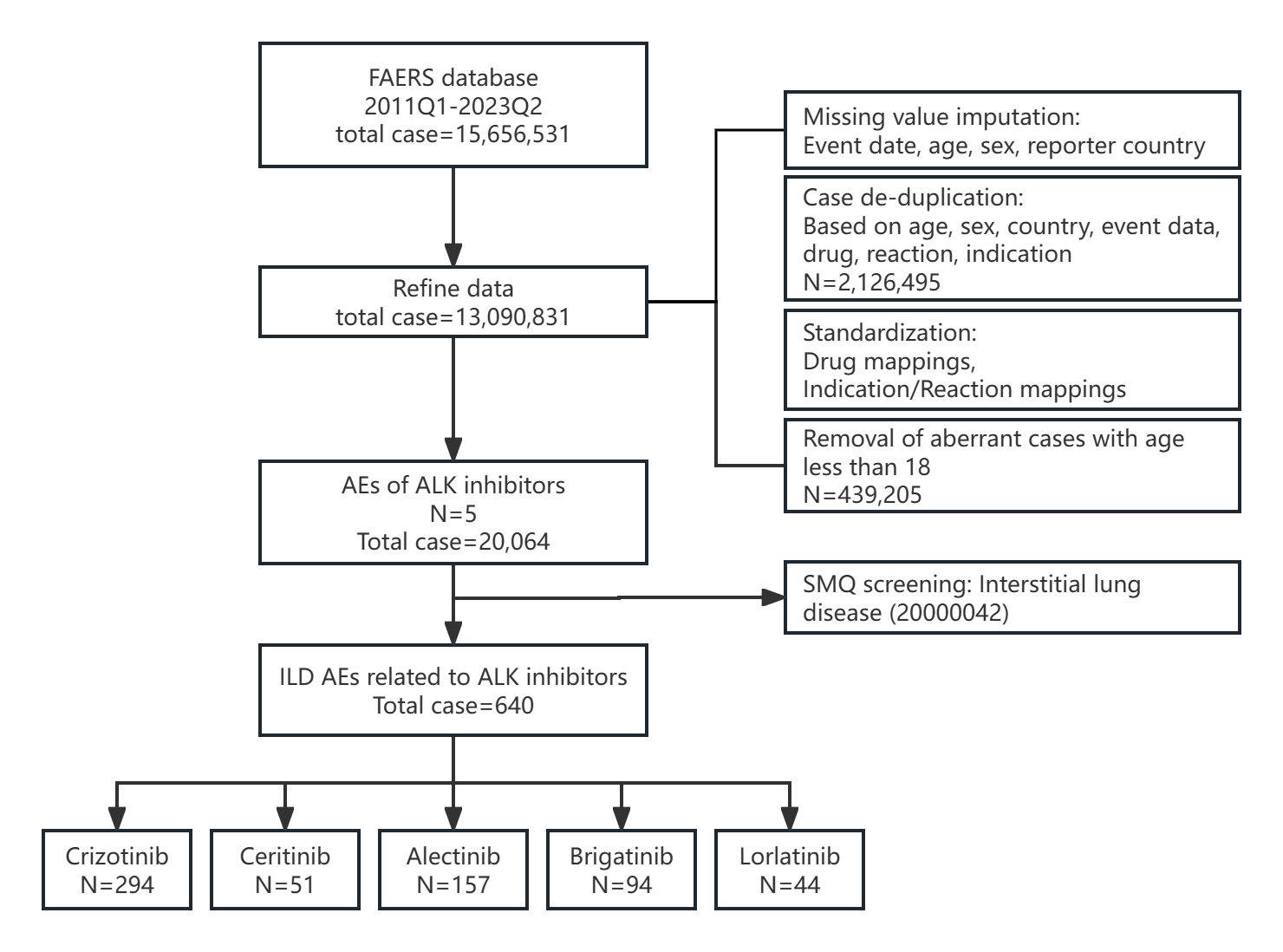
Supplementary Figure 1** Flow chart of data processing and analysis.

## Supplementary Tables

**Supplementary Table 1** Full list of preferred terms of ILD according to standardized MedDRA Queries

| Acute interstitial pneumonitis |
| --- |
| Alveolar lung disease |
| Alveolar proteinosis |
| Alveolitis |
| Alveolitis necrotising |
| Autoimmune lung disease |
| Bronchiolitis |
| Bronchiolitis obliterans syndrome |
| Chronic graft versus host disease in lung |
| Combined pulmonary fibrosis and emphysema |
| Confirmed e-cigarette or vaping product use associated lung injury |
| Diffuse alveolar damage |
| Eosinophilia myalgia syndrome |
| Eosinophilic granulomatosis with polyangiitis |
| Eosinophilic pneumonia |
| Eosinophilic pneumonia acute |
| Eosinophilic pneumonia chronic |
| Hypersensitivity pneumonitis |
| Idiopathic interstitial pneumonia |
| Idiopathic pneumonia syndrome |
| Idiopathic pulmonary fibrosis |
| Immune-mediated lung disease |
| Interstitial lung abnormality |
| Interstitial lung disease |
| Low lung compliance |
| Lung infiltration |
| Lung opacity |
| Necrotising bronchiolitis |
| Obliterative bronchiolitis |
| Pleuroparenchymal fibroelastosis |
| Pneumonitis |
| Probable e-cigarette or vaping product use associated lung injury |
| Progressive massive fibrosis |
| Pulmonary fibrosis |
| Pulmonary necrosis |
| Pulmonary radiation injury |
| Pulmonary toxicity |
| Pulmonary vasculitis |
| Radiation alveolitis |
| Radiation bronchitis |
| Radiation fibrosis - lung |
| Radiation pneumonitis |
| Respiratory syncytial virus bronchiolitis |
| Rheumatoid arthritis-associated interstitial lung disease |
| Small airways disease |
| Transfusion-related acute lung injury |
| Acute lung injury |
| Acute respiratory distress syndrome |
| Airway remodelling |
| Allergic eosinophilia |
| Antisynthetase syndrome |
| Biopsy lung abnormal |
| Complications of transplanted lung |
| Cystic lung disease |
| Goodpasture's syndrome |
| Granulomatosis with polyangiitis |
| Granulomatous pneumonitis |
| Langerhans' cell histiocytosis |
| Loefgren syndrome |
| Lung induration |
| Lung transplant rejection |
| Lupus pneumonitis |
| Lymphangioleiomyomatosis |
| Organising pneumonia |
| Pneumonitis chemical |
| Polyarteritis nodosa |
| Pulmonary alveolar haemorrhage |
| Pulmonary bullae rupture |
| Pulmonary contusion |
| Pulmonary eosinophilia |
| Pulmonary granuloma |
| Pulmonary haemosiderosis |
| Pulmonary renal syndrome |
| Pulmonary sarcoidosis |
| Pulmonary septal thickening |
| Restrictive pulmonary disease |
| Rheumatoid lung |
| Sarcoidosis |
| Systemic sclerosis pulmonary |
| Toxic oil syndrome |

**Supplementary Table** **2** Summary and AEs reports of FDA-approved ALK inhibitors.

| Generic name | Brand name | Aprroval year | Reports(N) |
| --- | --- | --- | --- |
| Crizotinib | Xalkori | 2011 | 8943 |
| Ceritinib | Zykadia | 2014 | 1905 |
| Alectinib | Alecensa | 2015 | 4651 |
| Brigatinib | Alunbrig | 2017 | 2133 |
| Lorlatinib | Lorbrena | 2018 | 2432 |

**Supplementary Table** **3** Number of reported ILD associated with ALK inhibitors among different time of onset.

| Drug  onset time | 0-30 | 31-60 | 61-90 | 91-180 | 181-360 | unknown |
| --- | --- | --- | --- | --- | --- | --- |
|  | (n) | (n) | (n) | (n) | (n) | (n) |
| ALK inhibitors | 179 | 43 | 29 | 50 | 14 | 325 |
| Crizotinib | 88 | 24 | 16 | 20 | 7 | 139 |
| Ceritinib | 13 | 0 | 1 | 5 | 3 | 29 |
| Alectinib | 32 | 12 | 7 | 18 | 3 | 85 |
| Brigatinib | 38 | 4 | 2 | 4 | 1 | 45 |
| Lorlatinib | 8 | 3 | 3 | 3 | 0 | 27 |

**Supplementary Table** **4** Univariate analysis with logistic regression analysis for the onset of ILD.

| **Background factor** | **With ILD(%)** | **Without ILD(%)** | **OR (95% CI)** | **P value** |
| --- | --- | --- | --- | --- |
| **Sex** |  |  | 0.76(0.65-0.90) | 0.0014 |
| Male | 289(50.2) | 9729(56.9) |  |  |
| Female | 287(49.8) | 7376(43.1) |  |  |
| **Age** |  |  | 1.03(0.86-1.23) | 0.76 |
| < 65 years old | 275(55.7) | 7188(56.4) |  |  |
| ≥65 years old | 219(44.3) | 5566(43.6) |  |  |
| **Concomitant diseases (diabetes, hypertension, dyslipidaemia, gastric disorder, constipation, pain)** |  |  | 2.17(1.65-2.85) | ＜0.001 |
| Yes | 582(90.9) | 15967(82.2) |  |  |
| No | 58(9.1) | 3456(17.8) |  |  |
| **Concomitant drug** |  |  | 2.45(2.08-2.87) | ＜0.001 |
| Yes | 279(43.6) | 4664(24.0) |  |  |
| No | 361(56.4) | 14759(76.0) |  |  |

**Supplementary Table 5** Full names and abbreviations in paper.

| **Full name** | **abbreviation** |
| --- | --- |
| Interstitial lung disease | ILD |
| Non-small-cell lung cancer | NSCLC |
| Standardized MedDRA Queries | SMQ |
| preferred term | PT |
| reporting odds ratio | ROR |
| proportional reporting ratio | PRR |
| information component | IC |
| empirical Bayes geometric mean | EBGM |
| tyrosine kinase inhibitor | TKI |
| proton pump inhibitors | PPI |
| anaplastic lymphoma kinase | ALK |
| Epidermal growth factor receptor | EGFR |
| ROS Proto-Oncogene 1 | ROS1 |
| mesenchymal-epithelial transition | MET |
| adverse events | AEs |
| adverse drug reaction | ADR |
